# CYP1B1 Knockout in a Bovine Hepatocyte-like Cell Line (BFH12) Unveils Its Role in Liver Homeostasis and Aflatoxin B1-Induced Hepatotoxicity

**DOI:** 10.3390/toxins17060294

**Published:** 2025-06-10

**Authors:** Silvia Iori, Ludovica Montanucci, Caterina D’Onofrio, Maija Lahtela-Kakkonen, Lorena Lucatello, Anisa Bardhi, Andrea Barbarossa, Francesca Capolongo, Anna Zaghini, Marianna Pauletto, Mauro Dacasto, Mery Giantin

**Affiliations:** 1 Department of Comparative Biomedicine and Food Science, University of Padua, 35020 Padua, Italy; silvia.iori@unipd.it (S.I.); caterina.donofrio@unipd.it (C.D.); lorena.lucatello@unipd.it (L.L.); francesca.capolongo@unipd.it (F.C.); marianna.pauletto@unipd.it (M.P.); mauro.dacasto@unipd.it (M.D.); 2 Department of Neurology, McGovern Medical School, UTHealth—University of Texas Health Science Centre at Houston, Houston, TX 77030, USA; ludovica.montanucci@uth.tmc.edu; 3 School of Pharmacy, University of Eastern Finland, 70210 Kuopio, Finland; maija.lahtela-kakkonen@uef.fi; 4 Department of Veterinary Medical Sciences, Alma Mater Studiorum University of Bologna, Via Tolara di Sopra 50, Ozzano dell’Emilia, 40064 Bologna, Italy; anisa.bardhi@unibo.it (A.B.); andrea.barbarossa@unibo.it (A.B.); anna.zaghini@unibo.it (A.Z.)

**Keywords:** CYP1B1, bovine, liver, AFB1, molecular docking, CRISPR/Cas9

## Abstract

CYP1B1 is a key enzyme involved in xenobiotic and endogenous metabolism, yet its physiological role in bovine liver homeostasis remains unclear. In this study, we generated a *CYP1B1* knockout (*CYP1B1*^KO^) bovine hepatocyte-like cell line to indirectly investigate its role in liver function. Transcriptomic analysis revealed alterations in immune regulation, epithelial barrier integrity, and detoxification pathways, with concurrent compensatory *CYP1A1* upregulation. Beyond its physiological role, CYP1B1 was found to actively participate in Aflatoxin B1 (AFB1) metabolism, a mycotoxin posing significant health risks to humans and livestock. Molecular docking suggested that CYP1B1 facilitates the conversion of AFB1 into AFM1 and AFBO. In agreement with these predictions, *CYP1B1*^KO^ cells exposed to AFB1 showed reduced AFM1 production and decreased cytotoxicity. Further transcriptomic analysis indicated that *CYP1B1*^KO^ cells exhibited mitigated oxidative stress and inflammatory responses, along with downregulation of *CYP3A74*, a key enzyme in AFB1 bioactivation. This suggests that *CYP1B1* KO reduces AFB1 toxicity by directly limiting AFB1 bioactivation and indirectly modulating the broader hepatic CYP network, further limiting the formation of toxic intermediates. These findings provide novel insights into *CYP1B1*’s function in bovine hepatocytes, highlighting its dual role in maintaining liver homeostasis and mediating AFB1 metabolism. The observed interplay between CYP1B1, CYP1A1, and CYP3A74 underscores the complexity of AFB1 biotransformation and warrants further investigation into the coordinated regulation of xenobiotic metabolism in cattle.

## 1. Introduction

Cytochrome P450 (CYP) enzymes play a crucial role in the metabolism of xenobiotics and endogenous compounds, influencing both bioactivation and detoxification processes [1,2,3]. Among them, CYP1B1 is involved in several key metabolic pathways, including the metabolism of steroid hormones, fatty acids, melatonin, and vitamins [4,5]. Although CYP1B1 is predominantly expressed in extrahepatic tissues such as the brain, kidney, and breast, its hepatic expression is limited but inducible, particularly upon exposure to aryl hydrocarbon receptor (AHR) ligands like 2,3,7,8-tetrachlorodibenzo-p-dioxin (TCDD) [6,7]. Additionally, CYP1B1 plays a prominent role in the activation of a variety of pro-carcinogens, including TCDD itself, polycyclic aromatic hydrocarbons (PAHs), heterocyclic amines, and 4-(methylnitrosamino)-1-(3-pyridyl)-1-butanone (NNK). It is also involved in the metabolism of endogenous 17β-oestradiol, producing oestradiol hydroquinones, which can be further converted into potentially carcinogenic semiquinones and quinones [5,8].

Despite this well-established involvement in xenobiotic metabolism and carcinogen activation, the physiological role of CYP1B1 in the liver—particularly in cattle—remains poorly understood.

Aflatoxins are secondary metabolites produced by fungi belonging to the *Aspergillus* genus, primarily *A. flavus* and *A. parasiticus*. These fungi contaminate a wide range of food and feed items, including corn, wheat, peanuts, milk, and eggs, thereby posing significant health risks to both humans and livestock, as well as causing substantial economic losses [9]. All aflatoxins (i.e., AFB1, AFB2, AFG1, AFG2), along with the AFB1-derived metabolite AFM1, have been classified as Group 1 carcinogens by the International Agency for Research on Cancer (IARC) [10]. Among them, AFB1 is considered the most dangerous, representing a particular threat to the liver due to its crucial role in mycotoxin metabolism [11].

In mammals and avian species, AFB1 metabolism is primarily mediated by CYP enzymes, which play a dual role in its bioactivation and detoxification. CYP1A and CYP3A subfamilies catalyse the formation of AFB1-8,9-epoxide (AFBO), a highly genotoxic intermediate capable of forming covalent adducts with DNA, RNA, and proteins, thereby damaging cellular macromolecules and contributing to mutations and carcinogenesis [12]. Additionally, CYP-mediated hydroxylation reactions generate AFM1 and the detoxified metabolite aflatoxin Q1 (AFQ1). AFB1 can also undergo reduction to aflatoxicol (AFL) via an NADPH reductase; this metabolite can be reconverted to AFB1, hence acting as a reservoir that extends AFB1’s persistence in the liver. Other detoxification routes include the CYP-mediated *O*-demethylation to form the relatively non-toxic aflatoxin P1 (AFP1), and AFB1 hydration, which yields AFB2a, a relatively non-toxic metabolite [11,13]. A key pathway to reduce the risk of AFBO-DNA adduct formation is the neutralization of AFBO with glutathione (GSH) through glutathione *S*-transferase (GST) enzymes [11]. AFBO can also be metabolized by microsomal epoxide hydrolase to form AFB1-dhd, a less toxic compound which can be further detoxified to the less reactive AFB1-dialcohol by Aldo-Keto Reductase Family 7 Member A3 (AFAR) [11,12].

Since AFB1 toxicity depends on the specific CYP isoforms expressed in a given species, variations in CYP-mediated metabolism and hepatic detoxification capacity strongly influence the extent of hepatotoxicity [14].

In humans, CYP1A2 and CYP3A4 are the primary hepatic enzymes responsible for AFB1 metabolism [15]. Additionally, CYP2A13 contributes to AFB1 metabolism in bronchial epithelial cells [16]. A recent in vitro study also identified CYP1B1 as being involved in AFB1 metabolism [17]. However, the role of CYP1B1 in AFB1 metabolism in cattle remains uncharacterized. AFB1 contamination in cattle feed is not rare. In Europe, some feed samples have shown alarming rates, with 27% testing positive for AFB1, and 18% exceeding the permitted maximum limit of 5 µg/kg for dairy cow feed [18,19].

Our previous study on AFB1 metabolism in bovine *CYP1A1*^KO^ and *CYP3A74*^KO^ hepatocyte-like cell lines indirectly suggested the pivotal role of CYP1A1 and CYP3A74 isoforms in AFB1 biotransformation/bioactivation and hepatotoxicity [20]. Here we investigated the function of *CYP1B1* in hepatocyte homeostasis and its specific role in AFB1 metabolism and hepatotoxicity. To achieve this, we established a new *CYP1B1*^KO^ bovine hepatocyte-like cell line, and we examined the physiological consequences of *CYP1B1* deletion in hepatocytes through transcriptomic analysis. We combined homology modelling and molecular docking with in vitro validation using the *CYP1B1*^KO^ cells to elucidate the role of *CYP1B1* in AFB1 biotransformation.

## 2. Results

### 2.1. Generation and Characterization of BFH12 CYP1B1^KO^ Cell Line

We used the RNP-complex approach to knock out the *CYP1B1* gene in BFH12 cells, achieving a transfection efficiency of ~90% (Supplementary Figure S1b). PCR amplification and Sanger sequencing of the targeted genomic region enabled the selection of a *CYP1B1*^KO^ clone for the subsequent stages of our study.

*CYP1B1* mRNA and protein amounts were assessed using qPCR and immunoblotting assays, respectively. Engineered cells exhibited a significant reduction in CYP1B1 expression at both the mRNA (~70%; *p* < 0.01) and protein (~60%; *p* < 0.01) levels compared to *CYP1B1*^CTL^ cells (Supplementary Figure S2a–c).

### 2.2. Transcriptome Analysis of BFH12 CYP1B1^KO^ Cells

A total of 663 differentially expressed genes (DEGs) were identified, of which 327 were upregulated and 336 were downregulated in *CYP1B1*^KO^ vs. *CYP1B1*^CTL^ cells (Supplementary Table S1).

### 2.3. Protein-Protein Interaction Network (PPI)

**Figure 1:**
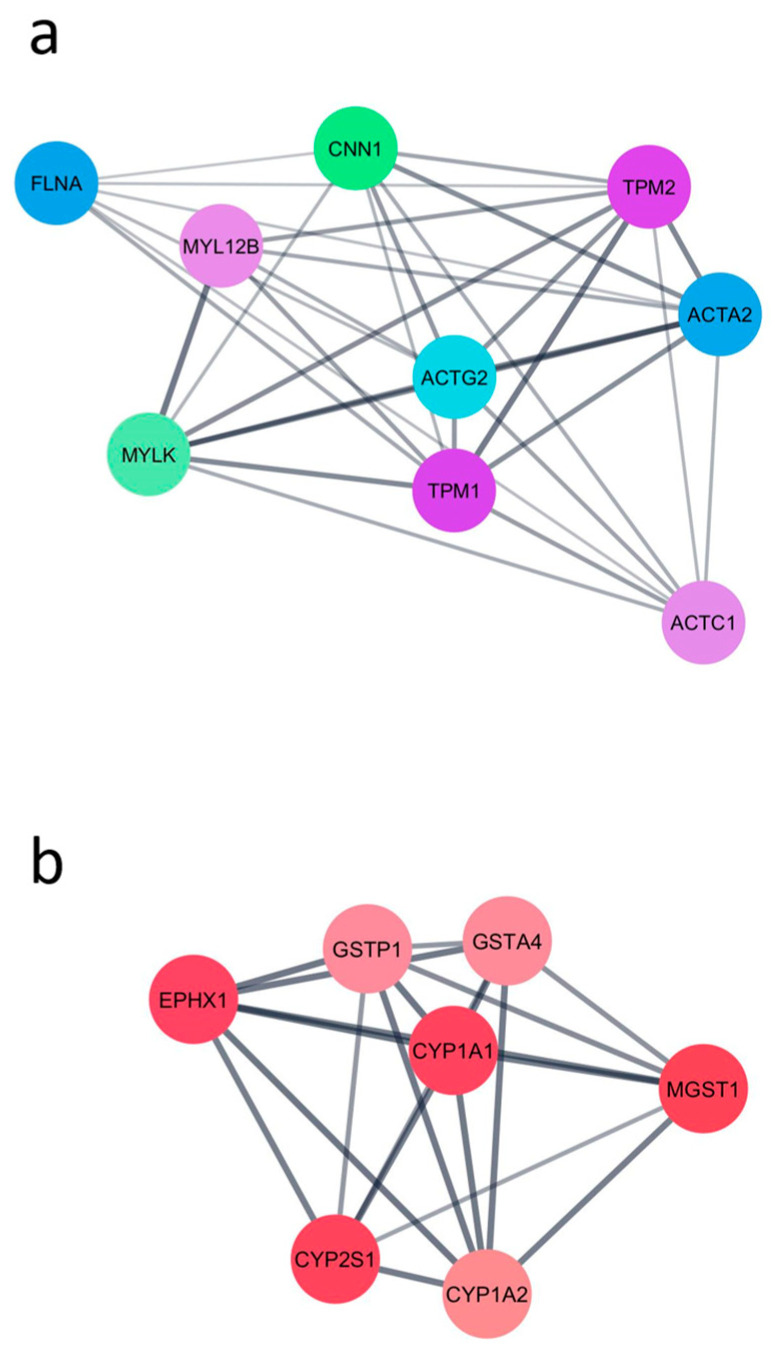
The two highest-scoring PPI modules obtained using the DEGs identified from the comparison between *CYP1B1*^KO^ and *CYP1B1*^CTL^ cells. (**a**) Module enriched in cytoskeleton-associated genes. (**b**) Module enriched in xenobiotic metabolism-related genes.

To identify functional gene modules impacted by *CYP1B1* KO, a PPI network was constructed using the DEGs identified from the comparison between *CYP1B1*^KO^ and *CYP1B1*^CTL^ cells. A total of 19 clusters were identified, indicating a high degree of functional interconnectivity among the DEGs. The two highest-scoring modules (with MCODE scores of 8.0 and 7.0, respectively) are shown in Figure 1. Module 1 (Figure 1a) included the genes Tropomyosin alpha-1 chain (*TPM1*), Myosin light chain kinase (*MYLK*), Actin, alpha 2, smooth muscle, aorta (*ACTA2*), Calponin 1 (*CNN1*), Myosin light chain 12B (*MYL12B*), Actin, gamma 2, smooth muscle (*ACTG2*), Filamin A (*FLNA*), and Actin, alpha cardiac muscle 1 (*ACTC1*), all primarily associated with cytoskeletal structure and contractile functions. Module 2 (Figure 1b) included Microsomal glutathione S-transferase 1 (*MGST1*), Epoxide hydrolase 1 (*EPHX1*), Glutathione S-transferase Pi 1 (*GSTP1*), Glutathione S-transferase Alpha 4 (*GSTA4*), Cytochrome P450 family 1 subfamily A member 1 (*CYP1A1*), Cytochrome P450 family 1 subfamily A member 2 (*CYP1A2*), and Cytochrome P450 family 2 subfamily S member 1 (*CYP2S1*), which are primarily involved in xenobiotic metabolism and detoxification pathways.

### 2.4. Gene Set Enrichment Analysis

Gene Set Enrichment Analysis (GSEA) was performed using the KEGG database to explore the transcriptional reprogramming induced by *CYP1B1* deletion in BFH12 cells under basal conditions. The top five significantly enriched gene sets (GSs) are shown in Supplementary Figure S3. The GS ‘Metabolic pathways’ was the most significantly enriched, encompassing numerous enzymes and transporters involved in the metabolism of endogenous and xenobiotic compounds, such as Malic enzyme 1 (*ME1*) and serine hydroxymethyltransferase 2 (*SHMT2*). The ‘Hypertrophic cardiomyopathy’ pathway was also significantly enriched, including genes such as ATPase sarcoplasmic/endoplasmic reticulum Ca^2+^ transporting 2 (*ATP2A2*) and others already identified in the PPI network (e.g., *TPM1* and *ACTC1*). The GS ‘Cytoskeleton in muscle cells’ included upregulated genes such as Caldesmon 1 (*CALD1*), Actin gamma 1 (*ACTG1*), and the previously mentioned *MYL12B*. The enrichment of the ‘Regulation of actin cytoskeleton’ GS was consistent with findings from the PPI network and comprised genes like Integrin subunit alpha 5 (*ITGA5*), Talin 2 (*TLN2*), and Filamin C (*FLNC*). Lastly, the GS ‘Nucleocytoplasmic transport’ included components of the nuclear pore complex and associated factors, such as Nucleoporin 93 (*NUP93*), Nucleoporin 188 (*NUP188*), and Importin subunit alpha 3 (*KPNA4*). The complete list of enriched GSs and contributing genes is provided in Supplementary Table S2.

### 2.5. KEGG Overrepresentation Analysis

The functional analysis identified 10 enriched KEGG pathways (Supplementary Figure S4) primarily related to inflammation and immune response (e.g., ‘Cytokine-cytokine receptor interaction’, ‘Staphylococcus aureus’, ‘Complement and coagulation cascades’), and xenobiotic metabolism (e.g., ‘Metabolism of xenobiotic by cytochrome P450’). *CYP1B1* deletion led to the downregulation of the following genes: several components of the complement system (such as *C1r*, *C1s*, *C2*, *C3*, and *CFB*); two members of the Bovine Major Histocompatibility Complex II (*BOLA-DRB* and *BOLA-DQA1*); several C-X-C motif chemokine ligands (i.e., *CXCL3*, *CXCL5*, *CXCL9* and *CXCL11*); *IL33* and Inhibin subunit beta E (*INHBE*); and Claudin 1 (*CLDN1*) and Claudin 15 (*CLDN15*). Conversely, *CYP1B1* knockout led to the upregulation of Transforming growth factor beta 3 (*TGFB3*), TNF receptor superfamily member 9 (*TNFRSF9*), and two cathepsins (i.e., *CTSL* and *CTSV*); Bradykinin receptor B2 (*BDKRB2*) and Coagulation factor II thrombin receptor (*F2R*); Selectin P (*SELP*) and E (*SELE*); *JAM2* and *PVR* (encoding for cell adhesion molecules), Tropomyosin 2 (*TPM2*), and Laminin subunit alpha 1 (*LAMC1*). Regarding specifically drug metabolizing enzymes, the genetic modification resulted in the downregulation of Glutathione S-transferase alpha 2 (*GSTA2*) and *MGST1*, *EPHX1*, and Aldehyde dehydrogenase 3 family member B1 (*ALDH3B1*); conversely, *CYP1A1* and glutathione peroxidase 1 (*GPX1*) were upregulated.

### 2.6. Homology Modelling and Molecular Docking of AFB1 into Bovine CYP1B1 Model

Homology modelling successfully generated a bCYP1B1 model based on the human CYP1B1 template (PDB: 3PM0). The positioning of the co-crystallized ligand in the bovine model confirms the reliability of the model for docking investigations.

**Figure 2:**
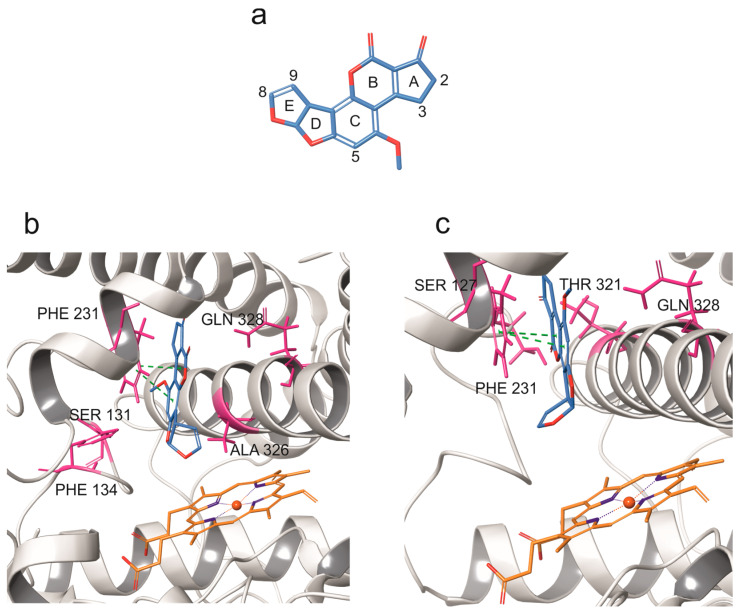
Molecular docking of AFB1 into bovine CYP1B1 model. (**a**) AFB1 structure, and (**b**,**c**) docking poses of AFB1 against bovine CYP1B1 model. CYP backbone is depicted in grey ribbon, while hydrophobic residues surrounding AFB1 are shown in pink. Green dashes indicate π-π stacking interactions.

The docking of AFB1 onto the bCYP1B1 model resulted in two relevant binding poses, illustrated in Figure 2. In the first pose (Figure 2b; docking score-DS: −7.9 kcal/mol), AFB1 was located within the binding pocket in proximity of hydrophobic residues SER 131 and ALA 326. In this pose, the distance between its C8 and the heme iron was 4.7 Å and π-π stacking interactions between AFB1 and PHE 231 were observed. In the second pose (Figure 2c; DS: −6.5 kcal/mol), AFB1 was surrounded by hydrophobic residues SER 127, THR 321, and PHE 231 and its E-ring was parallel to the heme iron. In this second pose, the distance between C8 and the heme iron was 5 Å and π-π stacking interactions between AFB1 and PHE 231 were also observed.

### 2.7. LC-MS/MS Quantification of AFB1, AFM1, AFL, and AFQ1

**Figure 3:**
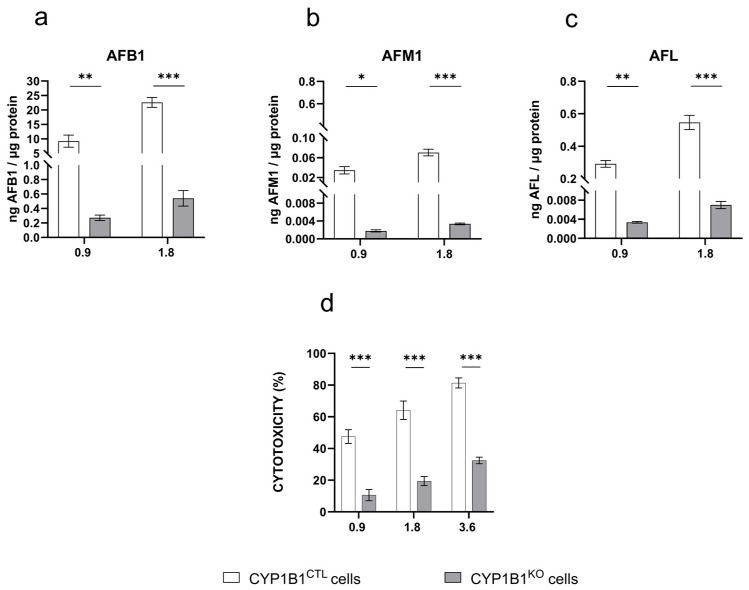
AFB1 metabolite pattern profile and cytotoxicity. Amount of (**a**) AFB1, (**b**) AFM1, and (**c**) AFL quantified by LC-MS/MS in the culture medium of *CYP1B1*^CTL^ and *CYP1B1*^KO^ cells treated with AFB1 0.9 and 1.8 µM. Data are expressed as ng of analyte per µg of total protein, as the mean ± mean standard error (SEM) of three biological replicates. (**d**) Cytotoxicity (WST-1 reagent assay) of increasing AFB1 concentrations in *CYP1B1*^KO^ and *CYP1B1*^CTL^ cells. Data are expressed as the mean percentage of dead cells relative to that of cells exposed to the vehicle only (0.1% DMSO) ± SEM of three biological replicates, each performed in sextuplicate. Statistical analysis: one-way ANOVA followed by Dunnett’s multiple comparisons test; *: *p* < 0.05, **: *p* < 0.01 and ***: *p* < 0.001.

Control cells treated with AFB1 produced detectable amounts of AFM1 and AFL that correlated with AFB1 concentration (Figure 3b,c). This confirms that BFH12 cells can metabolize AFB1 in a dose-dependent manner. In engineered cells, the amount of the parent compound as well as of AFM1 and AFL derivatives was significantly reduced (Figure 2a–c). The AFQ1 derivative was not detected in either control or genetically modified cells.

### 2.8. AFB1 Cytotoxicity

Upon AFB1 exposure, both *CYP1B1*^CTL^ and *CYP1B1*^KO^ cells exhibited a dose-dependent cytotoxic response. However, *CYP1B1*^KO^ showed a significant decrease in cell death at all the tested concentrations (approximately 40%, 45%, and 50% at 0.9, 1.8, and 3.6 µM AFB1, respectively; Figure 3d).

### 2.9. Transcriptome Analysis of BFH12 CYP1B1^KO^ Cells Exposed to AFB1

The comprehensive list of DEGs derived from the comparison between *CYP1B1*^KO^ and *CYP1B1*^CTL^ cells upon AFB1 exposure is provided in Supplementary Table S3. A total of 1958 and 2195 DEGs were identified at 0.9 and 1.8 μM, respectively. A Venn diagram was created to visualize the unique and shared DEGs following treatments with AFB1 at different concentrations (Supplementary Figure S5). The majority of DEGs (i.e., 86%) were modulated by the highest concentration of AFB1 (i.e., 1.8 μM). Therefore, the following functional analysis was conducted on DEGs resulting from the exposure to the highest AFB1 concentration, as the transcriptomic changes induced by this dose are expected to provide a more comprehensive overview of AFB1 effects on *CYP1B1*^KO^ cells.

### 2.10. PPI and Hub Gene Analysis Following AFB1 Exposure

The 2195 genes modulated by 1.8 μM AFB1 were analysed using eleven topological metrics implemented in CytoHubba. Genes were ranked, and the top 1% were selected as hub genes, resulting in the identification of 18 candidates. The highest-ranking node in the network was *PLK1* (Polo-like kinase 1), which displayed a cumulative centrality score of 55.4 and a downregulation pattern of expression. Among the other top-ranked genes, several were upregulated following AFB1 exposure, including B-cell lymphoma 2 (*BCL2*), Signal transducer and activator of transcription 1 (*STAT1*), E1A binding protein p300 (*EP300*), KRAS proto-oncogene, GTPase (*KRAS*), Cyclin D1 (*CCND1*), BRCA1 DNA repair associated (*BRCA1*), ATRX chromatin remodeller (*ATRX*), AT-rich interaction domain 4B (*ARID4B*), Diaphanous-related formin 3 (*DIAPH3*), and Phosphatidylinositol-4,5-bisphosphate 3-kinase catalytic subunit alpha (*PIK3CA*). In contrast, several key regulators were downregulated, including Interleukin 6 (*IL6*), CD44 molecule (*CD44*), Caveolin 1 (*CAV1*), CD74 molecule (*CD74*), Mitogen-activated protein kinase 3 (*MAPK3*), RuvB like AAA ATPase 2 (*RUVBL2*), and RNA polymerase II subunit C (*POLR2C*). The complete list of hub genes, including centrality scores and logFC, is reported in Supplementary Table S4.

### 2.11. GSEA upon AFB1 Treatment

GSEA was carried out on the full gene list derived from *CYP1B1*^KO^ cells exposed to 1.8 µM AFB1, using the KEGG database, to assess whether specific KEGG pathways showed coordinated transcriptional modulation. The top five enriched GSs are shown in Supplementary Figure S6. The GS ‘Coronavirus disease—COVID-19’ included several downregulated genes involved in innate immunity and inflammation, such as C-X-C motif chemokine ligand 8 (*CXCL8*) and toll like receptor 2 (*TLR2*). The ‘Ribosome’ GS included multiple downregulated structural ribosomal components, such as ribosomal protein S3 (*RPS3*), ribosomal protein L15 (*RPL15*), and ribosomal protein lateral stalk subunit P2 (*RPLP2*). In the ‘Oxidative phosphorylation’ GS, several genes involved in mitochondrial respiration were also downregulated, including NADH:ubiquinone oxidoreductase subunit B9 (*NDUFB9*) and ATP synthase membrane subunit c locus 1 (*ATP5MC1*). The ‘Focal adhesion’ GS contained genes such as integrin subunit alpha 5 (*ITGA5*), which was repressed upon AFB1 exposure, and laminin subunit beta 1 (*LAMB1*) and vascular endothelial growth factor A (*VEGFA*), both upregulated. Finally, the GS ‘Regulation of actin cytoskeleton’ included genes associated with structural dynamics, such as actinin alpha 1 (*ACTN1*), p21 (RAC1) activated kinase 1 (*PAK1*), and myosin heavy chain 10 (*MYH10*), that were upregulated upon AFB1 treatment. The full list of enriched GSs and associated genes is provided in Supplementary Table S5.

### 2.12. KEGG Overrepresentation Analysis of DEGs Modulated by AFB1

To isolate AFB1-specific transcriptional responses in the absence of CYP1B1, we performed KEGG pathway overrepresentation analysis on the set of DEGs obtained by excluding genes that were also differentially expressed in untreated *CYP1B1*^KO^ cells (i.e., 1891 out of 2195 DEGs). This approach allowed us to focus on AFB1-induced changes that are not attributable to the basal effects of *CYP1B1* deletion. Ten pathways (Supplementary Figure S7) were found to be enriched, reflecting an overrepresentation of genes associated with cell cycle regulation and proliferation (e.g., ‘Proteoglycans in cancer’, ‘Regulation of actin cytoskeleton’, ‘Prostate cancer’, Colorectal cancer’, ‘Renal cell carcinoma’, ‘AGE-RAGE signalling pathway in diabetic complications’). The 10 enriched pathways comprise several genes downregulated by AFB1 treatment, including Cyclin dependent kinase inhibitor 1A (*CDKN1A/P21*), Early growth response 1 (*EGR1*), Wnt family member 5A (*WNT5A*), and A-Raf proto-oncogene serine/threonine kinase (*ARAF*). Moreover, BCL2 associated X, apoptosis regulator (*BAX*), eukaryotic translation initiation factor 2 alpha kinase 1 (*EIF2AK1*), and eukaryotic translation initiation factor 2 subunit alpha (*EIF2S1*) were downregulated in *CYP1B1*^KO^ cells, along with mitogen-activated protein kinase 7 (*MAP3K7*) and 11 (*MAPK11*). The gene expression profile of *CYP3A74* resulted in being inhibited too. On the contrary, AKT serine/threonine kinase 3 (*AKT3*) and mechanistic target of rapamycin kinase (*MTOR*) were over-expressed in cells deprived of *CYP1B1*. The same pattern of expression was observed for Collagen type IV alpha 1 (*COL4A1*) and *COL4A2* chain genes.

### 2.13. Confirmatory qPCR Analyses

To validate the RNA-seq results, we quantified the mRNA expression of two representative DEGs for each part of the study using qPCR. For the characterization of the *CYP1B1*^KO^ model, *GPX1* and *GSTA2* were selected, while for the evaluation of AFB1 effects on *CYP1B1*^KO^ cells, *KRAS* and *NOX4* were analysed. As shown in Supplementary Figures S8 and S9, the qPCR data confirmed the RNA-seq findings, as both methods revealed consistent expression trends.

## 3. Discussion

### 3.1. The New Bovine CYP1B1^KO^ Hepatocyte-like Cell Line

Compared to our previous work on *CYP1A1* and *CYP3A74* KO cell lines, this study aims to better define the contribution of CYP1B1 to liver homeostasis and AFB1-induced transcriptional reprogramming. Unlike CYP1A1 and CYP3A74, which are well-characterized hepatic enzymes, CYP1B1 has been primarily considered an extrahepatic isoform with limited hepatic function. Our findings challenge this notion by demonstrating that CYP1B1 not only contributes to AFB1 metabolism but also exerts significant regulatory control in liver homeostasis. These insights add a previously unrecognized layer to the complexity of hepatic CYP-mediated responses and point to a distinct, non-redundant role of CYP1B1 in shaping the bovine liver’s response to xenobiotics.

To reach these goals, a new bovine *CYP1B1*^KO^ hepatocyte-like cell line was established using a CRISPR/Cas9 approach. Confirmatory pre-transcriptional and post-translational studies demonstrated an almost complete CYP1B1 ablation. A residual expression at both gene and protein level was expected, as contamination by non-homozygous cells, together with wild-type cells escaping clonal selection, has been previously observed in in vitro CRISPR/Cas9 applications [21,22,23].

### 3.2. Impact of CYP1B1 KO on BFH12 Cells Transcriptome: Effects on Cytoskeleton Organization, Xenobiotic Metabolism, and Immune Response

Transcriptomic profiling of *CYP1B1*^KO^ cells under basal conditions revealed an extensive transcriptional reprogramming, suggestive of a broad and multifaceted impact of CYP1B1 on hepatocyte physiology.

Functional PPI network analysis highlighted two major interconnected clusters of genes: one primarily composed of structural components involved in cytoskeletal organization and contractile function, and the other enriched in enzymes devoted to xenobiotic metabolism and detoxification [24,25,26,27,28,29,30,31,32,33]. This dual pattern points to concurrent alterations in cellular architecture and metabolic function. In line with these results, GSEA revealed a significant enrichment in GSs related to cytoskeletal remodelling, cellular metabolism, and nucleocytoplasmic transport, overall suggesting a global shift in metabolic processes, a state of enhanced cytoskeletal plasticity, and an adaptation to altered cellular stress responses.

KEGG enrichment analysis emphasized *CYP1B1* deletion-driven alterations in immune regulation further than in the abovementioned xenobiotic metabolism. Indeed, several components of the complement system (*C1r*, *C1s*, *C2*, *C3*, *CFB*) and chemokines (*CXCL3*, *CXCL5*, *CXCL9*, *CXCL11*) were repressed—consistent with our previous observations in *CYP1A1*^KO^ cells [21]—suggesting a convergent role of CYP1B1 and CYP1A1 in regulating immune homeostasis. The downregulation of *IL33* and *INHBE* highlights a reduction in pro-inflammatory signalling, indicating that CYP1B1 may influence inflammatory homeostasis even in the absence of exogenous toxins. The repression of MHC class II molecules (*BOLA-DRB* and *BOLA-DQA1*) indicates impaired antigen presentation and potential dampening of adaptive immunity. Finally, the upregulation of *TGFB3*, known for its role both in fibrosis and immune regulation [34], suggests a compensatory modulation of immune signalling, possibly counteracting the decreased inflammatory response.

CYP1B1 deletion also affected key detoxification pathways, embracing phase I and II drug-metabolizing enzymes. As an example, *CYP1A1* mRNA expression increased, indicating a potential compensatory mechanism that enhances *CYP1A1* expression to counteract the loss of CYP1B1. Given that both CYP1A1 and CYP1B1 are regulated by AHR, this adaptive response is not entirely unexpected and suggests a functional interplay between these two enzymes in maintaining metabolic homeostasis.

As to phase II metabolic enzymes, *CYP1B1* ablation caused the downregulation of *GSTA2* and *MGST1* conjugative enzymes [35], as well as *EPHX1*, a gene encoding for a crucial enzyme involved in the conversion of epoxides to dihydrodiols, which can then undergo further conjugation and excretion [36,37]. The same inhibitory effect was observed for *ALDH3B1*, which is involved in aldehyde detoxification, advising for an increased susceptibility to oxidative stress and toxic aldehyde accumulation, and consequently to a higher cellular susceptibility to toxic insults. A similar phenomenon was observed in *CYP1A1*^KO^ cells [21], suggesting that the loss of CYP1 enzymes may trigger common regulatory mechanisms affecting detoxification and oxidative stress pathways, and supporting the idea of a coordinated interplay between CYP1 enzymes and cellular defence mechanisms, which warrants deeper investigation.

Interestingly, *GPX1* exhibited increased expression exclusively in *CYP1B1*^KO^, whereas no such upregulation was observed in *CYP1A1*^KO^ cells [21]. This upregulation might represent a compensatory response to counteract oxidative stress, balancing the loss of the other detoxification enzymes. Particularly, such a selective upregulation of *GPX1* in CYP1B1-deleted cells, as confirmed by qPCR analysis, points to a putative distinct regulatory mechanism within this subfamily, underscoring the need for further investigation into the CYP1B1-specific role in oxidative stress and cellular defence.

Beyond detoxification, CYP1B1 deletion influenced epithelial barrier integrity, coagulation, and cellular signalling pathways. Indeed, *CLDN1* and *CLDN15*, encoding claudins essential for epithelial barrier integrity [38], were significantly downregulated, while *JAM2* and *PVR*, both adhesion-related molecules [39], were upregulated, indicating an increased vulnerability to barrier dysfunction and paracellular leakage, and a parallel compensatory mechanism toward the preservation of epithelial integrity. On the other hand, the induction of *BDKRB2* and *F2R* suggests potential alterations in haemostatic balance and vascular homeostasis, while the upregulation of *SELP* and *SELE* might reflect modifications in endothelial adhesion and immune cell trafficking, again underscoring the role of CYP1B1 in immune regulation.

Overall, these findings support a role for CYP1B1 in coordinating hepatic immune system, metabolic processes, oxidative stress defence, and barrier integrity under physiological conditions. Its deletion reveals a complex interplay of compensatory and disrupted pathways that extend beyond xenobiotic metabolism and underscore its importance in liver homeostasis.

### 3.3. CYP1B1 and AFB1: Molecular Docking and Metabolite Profiling in CYP1B1^KO^ Cells

The results of the molecular docking indicated that bCYP1B1 may be capable of harbouring the mycotoxin within its binding pocket. In particular, two binding poses were computationally predicted. In the first binding pose, the AFB1 *endo* side of the C8-C9 double bond was oriented toward the heme iron, suggesting the potential formation of the 8,9-*endo*-epoxide derivative. In the second binding pose, the production of both the AFM1 and the AFB1 8,9-*exo*-epoxide metabolites appeared to be more likely, as the E ring of AFB1 was oriented toward the porphyrin ring.

These computational predictions were here supported by in vitro experimental evidences, as a significant dose-dependent decrease in AFM1 production was observed in *CYP1B1*^KO^ cells. While this suggests a functional involvement of CYP1B1 in AFB1 metabolism, it does not directly confirm catalytic activity. However, these findings are consistent with previous evidences in humans, where CYP1B1 was proven to be implicated in AFB1 bioactivation [17].

Despite the absence of CYP1B1, AFB1 metabolism persisted in *CYP1B1*^KO^ cells, possibly due to the compensatory upregulation of *CYP1A1*, a known contributor to AFM1 synthesis in bovine hepatocytes [20]. This cellular response may help maintain some degree of AFB1 metabolism, leading to the residual formation of AFM1 observed in the KO cells. Moreover, the *CYP1A1* upregulation was further enhanced by AFB1 treatment, as previously observed in wild-type BFH12 cells [40]. Notably, in our previous study, CYP1A1 deletion completely abolished AFM1 production, strongly suggesting that CYP1A1 plays a dominant role over CYP1B1 in AFM1 formation [20].

As for AFL, its amount was significantly reduced in cells lacking CYP1B1. Since its formation is not CYP-mediated, this effect might be related to the downregulation of *AKR1A1*, as highlighted by RNA-seq. Indeed, this enzyme could be responsible for the reduction in AFB1 at the ketone carbonyl group, leading to AFL formation [13].

### 3.4. Reduced AFB1 Cytotoxicity and CYP3A74 Downregulation in CYP1B1^KO^ Cells

Cell viability analysis following AFB1 exposure revealed enhanced cellular resilience in the absence of CYP1B1. This indirectly implies a role for CYP1B1 in the conversion of AFB1 into genotoxic and hepatotoxic compounds, as suggested by molecular docking and LC-MS/MS analyses. To further explore the molecular mechanisms underlying CYP1B1-mediated AFB1 hepatotoxicity, we performed transcriptomic analysis by RNA-seq. Notably, we observed reduced expression of *CYP3A74* in *CYP1B1*^KO^ cells upon AFB1 exposure. As CYP3A74 is a key enzyme involved in AFB1 bioactivation to AFBO in bovine hepatocytes [20], this finding suggests that the diminished cytotoxicity in CYP1B1-deficient cells may result from both *CYP1B1* deletion and *CYP3A74* downregulation.

While these results are consistent with a role for CYP1B1 in AFB1 bioactivation, we fully acknowledge that they do not provide direct evidence of AFBO formation. The inability to detect AFBO is primarily due to the unavailability of analytical standards for AFBO and AFB1-dihydrodiol in LC-MS/MS workflows, and the high reactivity of AFBO with cellular macromolecules. This remains a limitation of the present study. Future investigations using orthogonal approaches—such as metabolite trapping or targeted profiling—will be required to conclusively establish the catalytic role of CYP1B1 in AFBO generation. The observed downregulation of *CYP3A74* in *CYP1B1*^KO^ cells raises intriguing questions regarding the interplay between these two enzymes. This phenomenon has already been brought to light in other species, where CYP enzyme crosstalk has been observed. Studies on rodents have shown that the deletion of one CYP enzyme can lead to altered expression levels of others, probably through transcriptional regulation via nuclear receptors such as PXR (pregnane X receptor) and CAR (constitutive androstane receptor), both of which regulate CYP3A expression [41]. Given that AHR regulates CYP1B1 [42], it is plausible that its deletion affects nuclear receptor signalling, indirectly influencing CYP3A74 expression [43]. The reduction in *CYP3A74* expression in *CYP1B1*^KO^ cells could therefore reflect a broader impairment in AFB1 bioactivation pathways, reinforcing the idea that multiple CYP enzymes contribute to AFB1 metabolism in a tightly regulated manner.

### 3.5. Adaptive Transcriptional Responses in CYP1B^KO^ Cells upon AFB1 Exposure: Stress Attenuation, Cytoskeletal Remodeling, and Apoptosis Suppression

RNA-seq analysis identified over 2100 DEGs following exposure of *CYP1B1*^KO^ cells to 1.8 µM AFB1. To further interpret such transcriptional changes, we constructed a PPI network, identifying 18 hub genes. Among the top-ranked upregulated genes, we observed *PLK1*, *EP300*, *PIK3CA*, and *KRAS*, all functionally linked to pro-fibrotic signalling [44,45,46,47]. Conversely, a known anti-fibrotic gene, *CAV1*, was downregulated, reinforcing the presence of a transcriptional profile skewed toward a fibrosis phenotype [48,49]. These findings suggest that, contrary to observations in human and murine hepatic stellate cells—where CYP1B1 inhibition has been associated with reduced fibrotic activation [50]—CYP1B1 deletion in bovine hepatocyte-like cells may favour the emergence of a pro-fibrotic transcriptional program under AFB1 exposure. This apparent discrepancy highlights the importance of species- and cell type-specific regulatory mechanisms in hepatic fibrosis and xenobiotic response.

GSEA analysis supports changes in stress and immune signalling, as well as in extracellular matrix (ECM) remodelling and cell motility. Specifically, the repression of *CXCL8* and *TLR2* transcripts indicated a dampened innate immune response, while the downregulation of genes encoding ribosomal proteins (i.e., *RPS3*, *RPL15*, and *RPLP2*) suggested a reduced protein synthesis activity. Additionally, the inhibition of the mitochondrial genes *NDUFB9* and *ATP5MC1* pointed to possible shifts in oxidative phosphorylation. As for ECM remodelling, the upregulation of *COL4A1*, *ITGA5*, *LAMB1*, *VEGFA*, *ACTN1*, and *PAK1* genes was indicative of ECM organization and fibrotic tissue adaptation [51,52,53,54,55,56]. As a whole, these results align with a broader transcriptional adaptation toward stress attenuation. Indeed, the downregulation of genes associated with ribosomal and oxidative phosphorylation GSs suggests a coordinated suppression of translational and mitochondrial activity, possibly as a mechanism to limit ROS accumulation during AFB1 exposure. At the same time, the upregulation of cytoskeletal and ECM-related genes reflects a cellular shift toward structural resilience and survival signalling. This remodelling may also overlap with fibrotic processes, as supported by PPI results, positioning CYP1B1 deletion as a modulator of both metabolic stress and tissue remodelling.

To distinguish changes induced by AFB1 from those linked solely to *CYP1B1* KO, we excluded DEGs already modulated under basal KO conditions. KEGG overrepresentation analysis on the remaining DEGs (*n* = 1891) highlighted ten pathways, mainly related to cell cycle regulation, apoptosis, oxidative stress response, xenobiotic metabolism, and DNA damage repair, indicating a broad reprogramming of stress response and detoxification mechanisms.

The downregulated genes included *MAP3K7*, *MAPK3*, and *MAPK11*, which encode proteins crucial for the MAPK pathway, a highly conserved cascade of protein kinases regulating various cellular processes in response to external stimuli [57]. These kinases play critical roles in regulating the expression of transcription factors like *EGR1*, which is known for its involvement in stress responses induced by DNA damage and oxidative stress [58,59]. In our study, we observed the inhibition of *EGR1* expression in *CYP1B1*^KO^ cells exposed to AFB1. The reduced expression of *EGR1* may result from decreased AFB1 bioactivation, leading to lower levels of DNA damage and reduced MAPK pathway activation. This hypothesis is reinforced by the downregulation of *CDKN1A* (*P21*), a cyclin-dependent kinase inhibitor that mediates cell cycle arrest in response to DNA damage [60]. Normally, cells exposed to AFB1 show increased DNA adducts formation, resulting in P21 induction and cell cycle arrest either to facilitate DNA repair or induce apoptosis if the damage is too severe [60]. Once again, it seems that *CYP1B1* KO leads to a decrease in the metabolic activation of AFB1, resulting in a lower number of DNA adducts, less DNA damage, and, subsequently, a reduced *P21* response. Interestingly, *CYP3A74*^KO^ cells also exhibited *P21* downregulation [22], suggesting that both genetic modifications mitigate AFB1-induced DNA damage responses, albeit through potentially different mechanisms. These findings support the hypothesis that CYP1B1 deletion reduces AFB1 cytotoxicity, at least in part, by downregulating *CYP3A74* expression and thereby impairing the AFB1 bioactivation pathway.

Significant changes were also observed in the Integrated Stress Response (ISR), evidenced by the downregulation of *EIF2AK1* and *EIF2S1*. The ISR is activated under stress conditions and typically reduces protein synthesis while promoting stress-related responses, including apoptosis [61]. The reduced activation of the ISR pathway in *CYP1B1*^KO^ cells suggests that these cells experience less stress in response to AFB1 exposure, further underscoring the protective role of *CYP1B1* deletion.

In terms of mitochondrial apoptosis regulation, a shift favouring cell survival was observed. *BCL2*, an anti-apoptotic gene, was upregulated, while *BAX*, a pro-apoptotic gene, was significantly downregulated. This shift enhances cellular resistance to apoptosis and correlates with increased expression of *KRAS*, *AKT3*, and *MTOR*, which are key components of the KRAS/AKT/mTOR pathway that promotes survival and growth [62], and is consistent with a transcriptional reprogramming toward pro-fibrotic adaptation. This molecular adaptation highlights the resilience of *CYP1B1*^KO^ cells to AFB1-induced cytotoxicity.

An intriguing finding, confirmed by qPCR analysis, was the upregulation of *NOX4*, a key member of the NADPH oxidase family known to generate ROS in AFB1-induced oxidative stress [63]. This effect is potentially linked to the overexpression of *CYP1A1*, as both enzymes influence ROS metabolism. CYP1A1 can generate ROS as part of its metabolic processes, especially in the presence of xenobiotics. On the other hand, NOX4 is a major source of ROS production within cells. Upregulation of *CYP1A1* might lead to increased ROS production, which could potentially influence the expression or activity of NOX4 as part of the cellular response to oxidative stress. Thus, among the numerous signalling pathways regulating the redox status, the AHR/NOX4 axis has recently been discovered [64].

Lastly, genes involved in the inflammatory response to AFB1, namely *TLR2* and *IL6*, were downregulated in *CYP1B1*^KO^ cells, as also observed in *CYP1A1*^KO^ cells [21]. This suggests that the deletion of CYP1B1, similarly to CYP1A1 deletion, reduces TLR2 activation, leading to decreased inflammation and hepatotoxicity.

### 3.6. Pros and Cons of the Study

Collectively, the transcriptomic outcomes here obtained underscore the multifaceted protective effects of CYP1B1 deletion, revealing how its absence indirectly influences DNA repair, oxidative stress, apoptosis, and inflammatory pathways, ultimately mitigating AFB1-induced cellular damage. Moreover, these findings highlight the intricate regulatory crosstalk among CYP enzymes and reinforce the concept that AFB1 metabolism in bovine hepatocytes is orchestrated by a tightly coordinated enzymatic network, wherein each CYP enzyme plays a distinct role in bioactivation and detoxification processes.

Recent advances in mammalian mechanistic toxicology have revealed that CYP enzymes are subject to intricate cross-regulatory networks mediated by transcriptional feedback loops and epigenetic controls, structural interaction, and redox partner modulation (e.g., P450 oxidoreductase) [65,66,67]. These mechanisms allow for dynamic responses to endogenous and exogenous signals, contributing to cellular adaptability and cross-regulation among CYP isoforms. Particularly, such regulation is highly tissue- and species-specific, influencing xenobiotic metabolism, physiological homeostasis, and disease susceptibility [66,68,69,70,71]. In the context of AFB1 metabolism, it has been reported that cross-regulation among CYP isoforms plays a central role in determining the balance between bioactivation and detoxification pathways [12,71,72,73,74]. Such a cross-regulation may induce systemic metabolic perturbations, particularly in lipid metabolism, which contribute to its hepatotoxicity [75,76].

Our findings—particularly the upregulation of *CYP1A1* and the downregulation of *CYP3A74* in *CYP1B1*^KO^ cells—underscore the presence of inter-enzyme crosstalk, suggesting that CYP1B1 modulates AFB1 metabolism not only directly but also indirectly by reshaping the broader hepatic CYP network.

Despite these advances, some limitations must be acknowledged.

AFBO could not be directly detected in our LC-MS/MS analyses, likely due to its high reactivity and short half-life. To overcome this limitation, future studies may employ metabolite trapping or derivatization techniques, which could enhance the stability and detectability of this key metabolite.

The use of a single CYP1B1 knockout clone limits the generalizability of our findings and does not allow us to rule out potential clonal variation or off-target effects. To address this, future validation studies should include multiple independent KO clones as well as non-manipulated wild-type controls.

Lastly, the inability of control cells to produce AFQ1 may indicate that BFH12 cells are not fully metabolically competent. In the future, the development of bovine liver organoids, which replicate the liver’s architecture and cellular diversity more accurately [77], could address this limitation.

## 4. Conclusions

This study offers novel insights into the hepatic role of bovine CYP1B1, both under basal conditions and in response to AFB1 exposure, thereby addressing a significant gap in the understanding of bovine liver physiology and xenobiotic metabolism.

Under basal conditions in hepatocyte-like cells, CYP1B1 deletion led to extensive transcriptional reprogramming, affecting pathways related to cytoskeletal architecture, immune regulation, and detoxification processes, suggesting a crucial role of CYP1B1 in maintaining hepatic homeostasis beyond its enzymatic functions in xenobiotic metabolism. In response to AFB1, CYP1B1 knockout decreased AFB1-induced cytotoxicity both lowering AFM1 production and inducing a survival-oriented adaptive response, including stress attenuation, cytoskeletal remodelling, and the suppression of apoptotic pathways.

In conclusion, this study underscores for the first time the multifaceted role of CYP1B1 in bovine liver physiology and its involvement in AFB1 metabolism, highlighting the need for further research to fully elucidate its functions and interactions with other hepatic enzymes.

## 5. Materials and Methods

### 5.1. Reagents and Chemicals

Complete William’s E Medium, foetal bovine serum (FBS), penicillin/streptomycin solution, Lipofectamine™ CRISPRMAX™ Cas9 Transfection Reagent, Phire Hot Start II DNA Polymerase, LightCycler 480 PowerUp™ SYBR^®^ Green Master Mix, Qubit RNA Assay Kit, High Capacity cDNA Reverse Transcription Kit, SuperSignal^®^ West Pico chemiluminescence substrate, and BCA Assay Kit were purchased from Thermo Fisher Scientific (Milan, Italy). The Alt-R CRISPR/Cas9 system was from IDT (Tema Ricerca, Bologna, Italy). The DNeasy Blood & Tissue kit and RNeasy Mini kit were provided by Qiagen (Milan, Italy). DNA, RNA, and the Protein Purification kit was from Macherey-Nagel (Düren, Germany). Rabbit anti-human beta-actin (ACTB, GTX109639) and the peroxidase-conjugated goat anti-rabbit IgG were from GeneTex (Prodotti Gianni, Milan, Italy). Rabbit anti-human CYP1B1 (A1377) was from ABclonal (Aurogene Srl, Rome, Italy). Analytical standards of AFB1, AFL, AFM1, AFQ1, and Aflatoxin M2 (AFM2, used as internal standard) were from TRC Canada (North York, ON, Canada). All plasticware used in cell culture and molecular biology experiments was from Sarstedt (Nümbrecht, Germany). All the other reagents were from Sigma Aldrich (Milan, Italy).

### 5.2. Generation and Characterization of BFH12 CYP1B1^KO^ Cell Line

The BFH12 cell line was genetically edited using the Alt-R CRISPR/Cas9 system. Guide RNAs (gRNAs) targeting exon 2 of the *CYP1B1* gene were designed with the CRISPOR tool (https://crispor.gi.ucsc.edu/; accessed in March 2023) (Supplementary Figure S1a, Supplementary Table S6). Cells were transfected with the ribonucleoprotein (RNP) complex composed of gRNAs and the Cas9 protein using the Lipofectamine™ CRISPRMAX™ reagent, and transfection efficiency was assessed by a flow cytometry analysis (CyFlow Space flow cytometer, Partec-System, Sysmex Europe GmbH, Norderstedt-Hamburg, Germany). Two days post-transfection, cells were diluted in culture medium, and individual clones were isolated, expanded, and screened for successful gene editing. Genomic DNA from selected clones was analysed via PCR and Sanger sequencing to confirm the deletion of the target gene region (Supplementary Table S7), as previously described [21,22]. BFH12 cells transfected without the RNP complex (using only the transfection reagent) were used as a negative control, designated as *CYP1B1*^CTL^.

To validate the *CYP1B1* KO, the gene and protein expression levels of CYP1B1 were assessed using qPCR and immunoblotting, respectively.

As for gene expression, *CYP1B1*^KO^ and *CYP1B1*^CTL^ cells were seeded into 6-well plates at a density of 50 × 10^3^ cells per well. Three biological replicates (passages 28–30), each performed in duplicate, were used. Four days after plating, total RNA was extracted using the RNeasy Mini Kit and quantified with the Qubit RNA Assay Kit on a Qubit 2.0 Fluorometer (Thermo Fisher Scientific, Milan, Italy). For qPCR, 1 µg of RNA was reverse-transcribed using the High Capacity cDNA Reverse Transcription Kit according to the manufacturer’s instructions. Amplification was performed as previously described [21] using 300 nM forward and reverse primers (Supplementary Table S8) and 2.5 ng of cDNA. Gene expression levels were quantified using the ∆∆Ct method [78].

As for protein expression, *CYP1B1*^KO^ and *CYP1B1*^CTL^ cells (passages 28–30) were seeded in 90 mm Petri dishes at a density of 3 × 10^5^ cells per dish. Four days after plating, the culture medium was collected, and the cell monolayers were washed with hypotonic lysis buffer (20 mM Tris-HCl, pH 7.4) and scraped using 300 µL of the same buffer. The suspension underwent repeated freeze–thaw cycles, alternating between liquid nitrogen and a 37 °C water bath. Cell debris was removed by centrifugation (12,000× *g*, 10 min, 4 °C), and the supernatant was collected. Protein concentration was measured using the BCA assay Kit, following the manufacturer’s instructions. For the immunoblotting assay, 20 µg of total protein were loaded onto a minigel, followed by transfer onto nitrocellulose membranes as previously described [23,24]. The membrane was then incubated overnight with rabbit anti-human CYP1B1 primary antibody (dilution 1:1000) or for 2 h with anti-ACTB primary antibody (1:6000). This step was followed by the incubation with the secondary antibody, namely a horseradish peroxidase-conjugated goat anti-rabbit (1:6000) for 1.5 h. The immunopositive bands were finally semi-quantified using the method described in [79].

Finally, the transcriptome of *CYP1B1*^KO^ cells was fully characterized by means of RNA sequencing (see details below).

### 5.3. Homology Modelling and Molecular Docking of AFB1 into CYP1B1 Model

Molecular modelling was performed using Schrödinger Maestro version 12.8 (Small-Molecule Drug Discovery Suite 2021-2, Schrödinger, LLC, New York, NY, USA). Due to the absence of an experimentally determined bovine CYP1B1 crystal structure, the human CYP1B1 crystal structure complexed with α-naphthoflavone (PDB: 3PM0; resolution: 2.70 Å) [80] was chosen as the template and homology modelling was conducted. This structure was selected for its high-resolution quality and 80% sequence identity with bovine CYP1B1, making it a reliable model for structural prediction.

The template was firstly prepared with Protein Preparation Wizard; specifically, bond orders and hydrogens were added, and missing chains and missing loops were filled using Prime. Het states were generated using Epik (pH 7 ± 2). The heme iron was set as Fe^3+^ and it was connected via zero-order bonds to the cysteine sulphur and to the four heme nitrogens. The hydrogen bonds were assigned using PROPKA (pH 7.0) and waters having less than 3 H-bonds to non-waters were removed. The refinement module was used to perform restrained minimization using the OPLS2005 force field, with a heavy atom converging 0.30 Å RMSD.

Build Homology Model was used to obtain the 3D model of bCY1B1. Structurally Conserved Regions (SCRs) were checked and a Ramachandran plot was built to evaluate amino acids–outliers.

Co-crystalized ligand and AFB1 (PubChem CID: 186907) were prepared for dockings with the LigPrep tool. LigPrep was used to add hydrogens, set ionization (at pH 7 ± 2) and the tautomeric state using Epik, optimize the H-bonding network, and minimize ligand structures with the OPLS4 force field.

Molecular docking was performed using Glide, with a standard precision docking protocol. The grid box was centred on the active site, encompassing all residues within 20 Å of the heme group. A co-crystallized ligand of the template (i.e., α-naphthoflavone) was docked in the bCYP1B1 model to assure a correct binding mode for the target (i.e., AFB1). Then, the AFB1 ligand was docked into the model. Docking analyses were repeated multiple times (at least 3) to ensure consistency and reproducibility of the results. For each docking run, 10 binding poses were generated and evaluated based on their docking scores. The two most relevant poses, showing the lowest docking scores, were selected for further analysis.

### 5.4. Cells’ Incubation with AFB1 for Cytotoxicity, LC-MS/MS, and RNA-Seq Investigations

For cytotoxicity evaluation, *CYP1B1*^KO^ and *CYP1B1*^CTL^ cells (passages 28–30) were seeded in 96-well plates at a density of 6 × 10^3^ cells/well. Four days after plating, they were incubated for 48 h with AFB1 (0.9, 1.8, and 3.6 μM) dissolved in FBS-free medium containing 0.1% DMSO. Cells treated with 0.1% DMSO only served as the negative control. AFB1 concentrations were selected according to a previous dose–response curve [81] and aligned with those employed in earlier studies [20,40]. Each experimental group included three biological replicates, with each concentration tested in six separate wells.

For LC-MS/MS and RNA-seq investigations, *CYP1B1*^KO^ and *CYP1B1*^CTL^ cells (passages 28–30) were seeded in 6-well culture plates at a density of 5 × 10^4^ cells/well. Four days after plating, they were exposed to 0.9 and 1.8 μM AFB1 for 48 h. The medium was collected and stored at −80 °C, and the respective cell monolayers underwent total protein isolation as described in Section 5.2. Conversely, for total RNA isolation, an additional set of cell monolayers was washed, scraped off, and stored at −80 °C in RLT buffer (Qiagen) containing β-mercaptoethanol as previously described [20]. Total RNA was then isolated using the RNeasy Mini Kit.

### 5.5. LC-MS/MS Quantification of AFB1, AFM1, AFL and AFQ1

The amount of AFB1 and its metabolites (AFM1, AFL, and AFQ1) was measured in the medium of all experimental conditions by LC-MS/MS, as described elsewhere [22]. To evaluate the method’s performance in terms of linearity, precision, and accuracy, blank medium-based calibrators (six levels ranging from 0.1 to 20 ng/mL) and quality control (QC) samples (0.1, 1, and 10 ng/mL, in triplicate) were prepared on each day of analysis. The lower limit of quantification (LLOQ), defined as the lowest concentration detected with a signal-to-noise (S/N) ratio ≥10 and acceptable accuracy and precision (<20%) based on four replicate injections, was established at 0.1 ng/mL for all analytes. The method demonstrated excellent accuracy (consistently within ±15%) and precision (coefficient of variation below 15%) across all QC levels. The injection of blank matrix samples following the highest point of the calibration curves proved the specificity of the method, along with the absence of carry-over. Additionally, recovery for all compounds always fell within 80–120%, confirming the method’s reliability. Data obtained from sample analysis were expressed as the amount of metabolite in the culture medium divided by the total protein content of the respective cell monolayer. Three independent experiments (i.e., three different cell passages) were executed for each experimental condition.

### 5.6. RNA-Seq Analysis

Library construction using the QuantSeq 3′ mRNA-Seq Library Prep Kit FWD (Lexogen GmbH, Vienna, Austria) and sequencing with the Illumina Novaseq 6000 (single-end 75 bp) were conducted at the Next Generation Sequencing (NGS) facility of the Department of Biology at the University of Padua, Italy. A total of 12 tagged cDNA libraries were sequenced. Two experimental sets were analysed. The first set consisted of *CYP1B1*^KO^ and *CYP1B1*^CTL^ cells, with the aim of revealing the transcriptomic perturbation caused by the KO of the *CYP1B1* gene. The second set included *CYP1B1*^KO^ and *CYP1B1*^CTL^ cells treated with 0.9 and 1.8 µM AFB1, aiming to highlight changes induced by the mycotoxin exposure. For each experimental condition, three biological replicates were considered.

The obtained raw reads were trimmed using the BBDuk program (BBTools suite) and then mapped against the Bos taurus ARS-UCD1.2 reference genome as previously reported [20]. The differential expression analysis was conducted using the EdgeR Bioconductor package (v3.40.2) [82]. Specifically, pair-wise comparisons between *CYP1B1^KO^* and *CYP1B1^CTL^* samples were carried out to highlight transcriptional changes induced by CYP1B1 KO, setting a False Discovery Rate (FDR) of 5%. Then, pair-wise comparisons were carried out to highlight transcriptional changes induced by AFB1 exposure in *CYP1B1^KO^* and *CYP1B1^CTL^* cells (FDR < 0.05).

The ClusterProfiler package (v4.10.0) [83] was then implemented in R environment to functionally interpret differentially expressed genes (DEGs) through Gene Set Enrichment Analysis (GSEA) and the KEGG overrepresentation test, as described in [83].

### 5.7. PPI Network and Hub Genes’ Analyses

The PPI network was constructed using the online database Search Tool for the Retrieval of Interacting Genes/Proteins (STRING) with default parameters (https://string-db.org/cgi/input.pl, accessed on 7 April 2025) and visualized in Cytoscape (version 3.10.3; https://cytoscape.org/, accessed on 7 April 2025). The Cytoscape plugin MCODE was implemented to detect modules with the following parameters: Degree cutoff = 10, Node Score cutoff = 0.2 and K-Core = 2.

To identify key regulatory genes within the PPI network upon AFB1 exposure, eleven topological metrics implemented in the CytoHubba plugin (i.e., MCC, MNC, EPC, Degree, Closeness, Radiality, Stress, Betweenness, BottleNeck, DMNC, and EcCentricity) were computed for each node. All metrics were normalized and combined to derive an aggregate centrality score. Genes were then ranked based on this composite score, and the top 1% were selected as hub genes. This integrative approach allows for a more robust and comprehensive identification of functionally central nodes, minimizing bias associated with any single metric.

### 5.8. Confirmatory qPCR Analysis

Four target transcripts—*GPX1* and *GSTA2* (modulated by *CYP1B1* KO), and *KRAS* and *NOX4* (modulated by AFB1 exposure in *CYP1B1*^KO^ cells)—along with one internal control gene (*RPLP0*), were selected for qPCR validation of the RNA-seq data. Gene-specific primers (Supplementary Table S8) were designed using the Primer-BLAST web service (https://www.ncbi.nlm.nih.gov/tools/primer-blast/; accessed on 16 May 2025). Primer specificity was evaluated either in silico by means of the BLAST tool (https://blast.ncbi.nlm.nih.gov/Blast.cgi; accessed on 25 May 2025) or experimentally by melting curve analysis. The same total RNA used in the RNA-seq experiments was employed for the confirmatory qPCR analyses. Reverse transcription (1 µg of total RNA) and qPCR amplification (1.25 ng cDNA per reaction) were performed as described above (Section 5.2). The quality of each qPCR assay was gathered from standard curve slopes (Supplementary Table S9). The PCR efficiency (E%) was calculated using the equation E = ((10^−1/slope^) − 1) × 100 and was considered acceptable with values between 90% and 110% (Supplementary Table S9). Gene expression levels were quantified using the ∆∆Ct method [78].

## Data Availability

Raw Illumina Sequencing Data have been deposited in GenBank (SRA) under the BioProjects IDs PRJNA1068490 and PRJNA1141944.

## Supplementary Materials

The following supporting information can be downloaded at: https://www.mdpi.com/article/10.3390/toxins17060294/s1, Table S1: DEGs between *CYP1B1*^KO^ and *CYP1B1*^CTL^ cells; Table S2: GSEA (KEGG pathways) under basal conditions; Table S3: DEGs between *CYP1B1*^KO^ and *CYP1B1*^CTL^ cells upon AFB1 exposure; Table S4: Network topology parameters of hub genes identified following AFB1 exposure; Table S5: GSEA (KEGG pathways) upon AFB1 treatment; Table S6: List of crRNAs used for CRISPR/Cas9-mediated KO of bovine CYP1B1 gene; Table S7: List of primers used for genotyping analysis; Table S8: List of primers used for qPCR analysis; Table S9: qPCR assay parameters; Figure S1: CRISPR/Cas9-mediated *CYP1B1* KO; Figure S2: CYP1B1 mRNA level and apoprotein amount in CTL and KO cells; Figure S3: GSEA (KEGG pathways) under basal conditions; Figure S4: KEGG overrepresentation analysis conducted on DEGs between *CYP1B1*^KO^ and *CYP1B1*^CTL^ cells; Figure S5: Venn diagram reporting number and percentages of unique and shared DEGs among the different AFB1 treatment conditions (i.e., 0.9 µM and 1.8 µM) in *CYP1B1*^KO^ vs. *CYP1B1*^CTL^ cells; Figure S6: GSEA analysis (KEGG pathways) following treatment with 1.8 µM AFB1; Figure S7: KEGG overrepresentation analysis conducted on DEGs between *CYP1B1*^KO^ and *CYP1B1*^CTL^ cells incubated with AFB1 1.8 µM; Figure S8: qPCR validation of two DEGs modulated by *CYP1B1* KO; Figure S9: qPCR validation of two DEGs modulated by AFB1 (0.9 and 1.8 µM) in *CYP1B1*^KO^ cells. Refs. [84,85,86,87,88] are cited in Supplementary Materials.
