# Supplementary material for: CYP1B1 Knockout in a Bovine Hepatocyte-like Cell Line (BFH12) Unveils Its Role in Liver Homeostasis and Aflatoxin B1-Induced Hepatotoxicity
Source: Toxins (Basel). 2025 Jun 10;17(6):294. doi: 10.3390/toxins17060294 (PMC12197428; doi:10.3390/toxins17060294)
Supplement: Supplementary file 1 [file toxins-17-00294-s001.zip › toxins-3544366-supp.pdf]

Supplementary Materials

CYP1B1 Knockout in a Bovine Hepatocyte-like cell line (BFH12) Unveils Its Role in Liver Homeostasis and Aflatoxin B1-Induced Hepatotoxicity

Silvia Iori [1], Ludovica Montanucci [2], Caterina D'Onofrio [1], Maija Lahtela-Kakkonen [3], Lorena Lucatello [1], Anisa Bardhi [4], Andrea Barbarossa [4], Francesca Capolongo [1], Anna Zaghini [4], Marianna Pauletto [1], Mauro Dacasto [1] and Mery Giantin [1,*]

Tables

Supplementary Table 6. List of crRNAs used for CRISPR/Cas9 mediated KO of bovine *CYP1B1* gene.

Guide ID	Sequence	Target region
CYP1B1#1	GTAGCGCGTAGGTCAGATCA	Promoter
CYP1B1#2	AATAGTACCTCACGAGCCCT	3′ UTR

Supplementary Table 7. List of primers used for genotyping analysis.

Primers ID	Sequence (5′ → 3′)
CYP1B1#FW	GTCGGGGGAATCGGGTCATA
CYP1B1#REV	GAGGCATTTCAGTAACCATGCC

Supplementary Table 8. List of primers used for qPCR analysis.

Target gene	Primer	Primer sequence 5′ → 3′	Amplicon Size	References
CYP1B1	Forward	CACCAGGTATTCGGAAGTGC	118	Girolami, 2011 [84]
	Reverse	AAGAAAGGCCATGACGTAGG		
RPLP0	Forward	CAACCCTGAAGTGCTTGACAT	227	Robinson, 2007 [85]
	Reverse	AGGCAGATGGATCAGCCA		
GPX1	Forward	GGGCATCAGGAAAACGCC	88	Lopparelli, 2012 [86]
	Reverse	GCATAAAGTTGGGCTCGAACC		
GSTA2	Forward	TTACCACTGTGCCCACCTGAT	112	Girolami, 2015 [87]
	Reverse	CTTGTCCGTGATTCTTCAGCAC		
KRAS	Forward	ACACAAAACAGGCTCAGGACT	92	Yu, 2022 [88]
	Reverse	GAAGGCATCGTCAACACCC		
NOX4	Forward	CCGCGAGGATTTTGTCGAAC	106	Designed *ex novo*
	Reverse	CAGTACCATGCAGACCCCTG		

[84] Girolami, F., Spalenza, V., Carletti, M., Perona, G., Sacchi, P., Rasero, R., & Nebbia, C. (2011). Gene expression and inducibility of the aryl hydrocarbon receptor-dependent pathway in cultured bovine blood lymphocytes. Toxicology letters, 206(2), 204-209. DOI: 10.1016/j.toxlet.2011.07.014

[85] Robinson, T. L., Sutherland, I. A., & Sutherland, J. (2007). Validation of candidate bovine reference genes for use with real-time PCR. Veterinary immunology and immunopathology, 115(1-2), 160-165. DOI: 10.1016/j.vetimm.2006.09.012

[86] Lopparelli, R. M., Giantin, M., Pozza, G., Stefani, A. L., Ravarotto, L., Montesissa, C., & Dacasto, M. (2012). Target gene expression signatures in neutrophils and lymphocytes from cattle administered with dexamethasone at growth promoting purposes. Research in veterinary science, 93(1), 226-233. DOI: 10.1016/j.rvsc.2011.07.004.

[87] Girolami, F., Spalenza, V., Manzini, L., Carletti, M., & Nebbia, C. (2015). Constitutive expression of the AHR signaling pathway in a bovine mammary epithelial cell line and modulation by dioxin-like PCB and other AHR ligands. Toxicology Letters, 232(1), 98-105. DOI: 10.1016/j.toxlet.2014.09.013

[88] Yu, W., Jiang, H., Liu, F., Li, Z., Xu, L., Liu, C., ... & Yuan, B. (2022). KRAS Affects the Lipid Composition by Regulating Mitochondrial Functions and MAPK Activation in Bovine Mammary Epithelial Cells. Animals, 12(22), 3070. DOI: 10.3390/ani12223070.

Supplementary Table 9. qPCR assay parameters.

Target gene	Primer concentration (nM)	Efficiency (%)	Error	Dynamic range
CYP1B1	300F/300R	96.0	0.011	22.64 – 34.37
RPLP0	300F/300R	96.0	0.003	15.31 - 27.11
GPX1	300F/300R	92.3	0.022	22.42 - 32.87
GSTA2	300F/300R	101.2	0.002	20.76 - 30.80
KRAS	300F/300R	101.0	0.014	21.21 - 31.83
NOX4	300F/300R	102.6	0.061	25.12 - 33.79

Figures

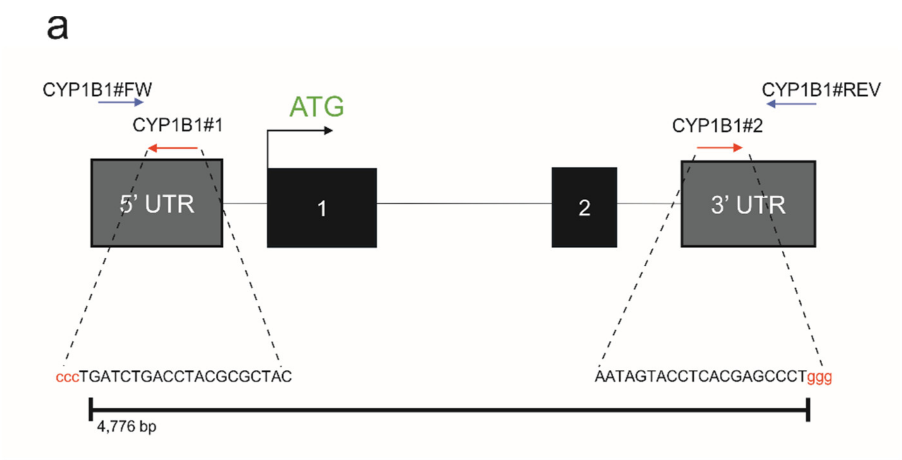

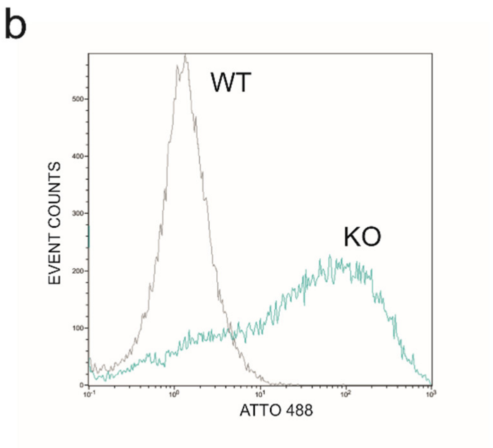

Cells	% Transfected cells
WT	0
KO	90

Supplementary Figure 1. CRISPR/Cas9-mediated *CYP1B1* KO. (**a**) gRNAs (indicated by red arrows) designed to guide the Cas9 machinery to the *CYP1B1* promoter region and 3' UTR. Cas9 activity at these sites results in a double-stranded break, leading to a deletion of approximately 4,776 bp. Primers (indicated by blue arrows) flanking the deleted DNA region used to in the PCR-based confirmation of the correct deletion. (**b**) Flow cytometry analysis of (KO green) and native wild-type (WT, grey) cells.

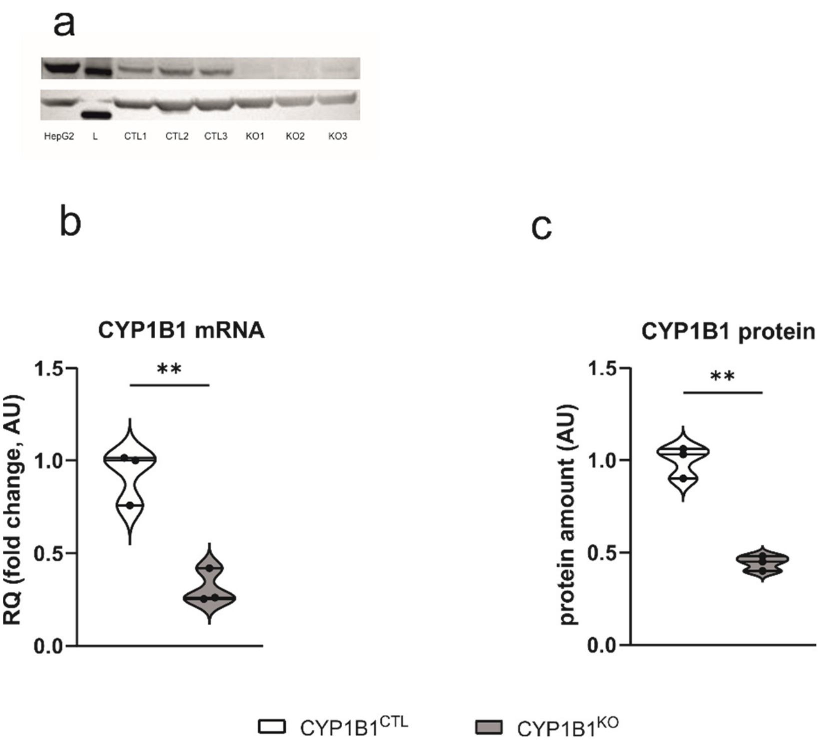

Supplementary Figure 2. CYP1B1 mRNA level and apoprotein amount in CTL and KO cells. (a) Gene expression data (relative quantification, RQ, fold change, arbitrary units, AU) are reported as the mean ± SEM of three biological replicates (each performed in duplicate)**.** (**b**) CYP1B1 immunoblotting; BACT was used as the loading control and HepG2 as the positive control. (**c**) Densitometric analysis of CYP1B1 immunoblotting; data are expressed in AU as the mean ± SEM of three biological replicates. Statistical analysis: unpaired t-test with Welch's correction. **: $p < 0.01$, *CYP1B1*^{KO} *vs* *CYP1B1*^{CTL} cells.

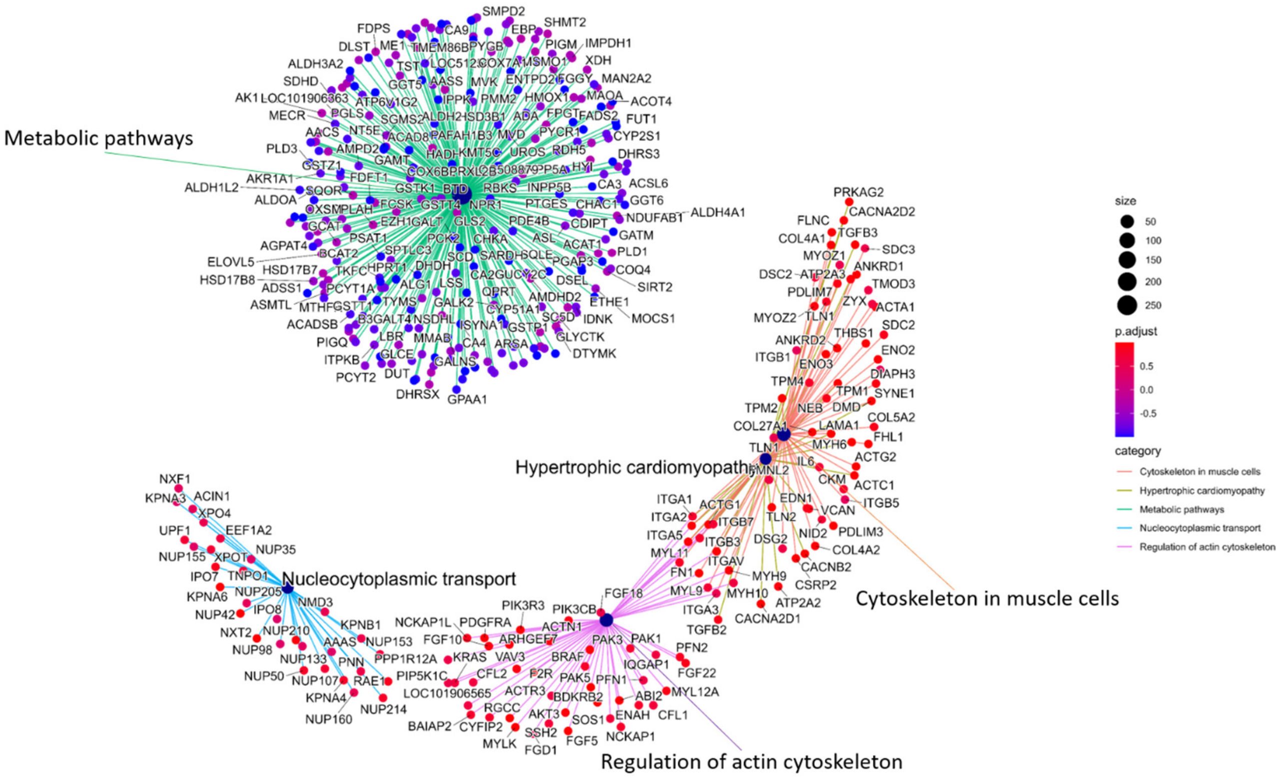

Supplementary Figure 3. GSEA analysis (KEGG pathways) under basal conditions. The network plots (cnetplots) display the top five significantly enriched pathways. Node colors represent the adjusted p-values (p.adjust), calculated using the Benjamini–Hochberg (BH) correction method. Genes are connected to the corresponding pathways based on enrichment results.

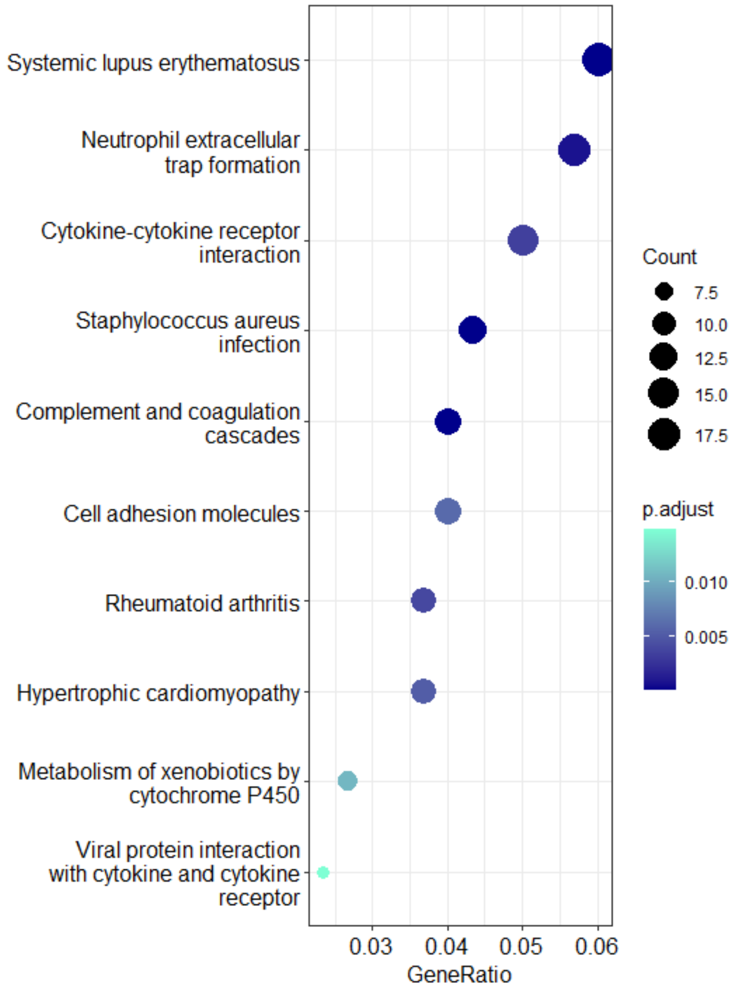

Supplementary Figure 4. KEGG overrepresentation analysis conducted on DEGs between *CYP1B1*^{KO} and *CYP1B1*^{CTL} cells. Count (dot size) represents the number of DEGs enriched in a certain pathway. The colour gradient represents the adjusted significance level (p. adjusts), according to the BH method. The Gene Ratio represents the proportion between the number of DEGs enriched in a given pathway and the total number of genes annotated in that pathway.

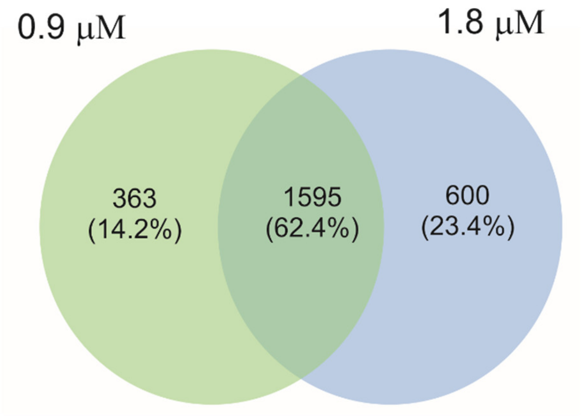

Supplementary Figure 5. Venn diagram reporting number and percentages of unique and shared DEGs among the different AFB1 treatment conditions (i.e., 0.9 μM and 1.8 μM) in $\textit{CYP1B1}^{\mathrm{KO}}$ vs $\textit{CYP1B1}^{\mathrm{CTL}}$ cells.

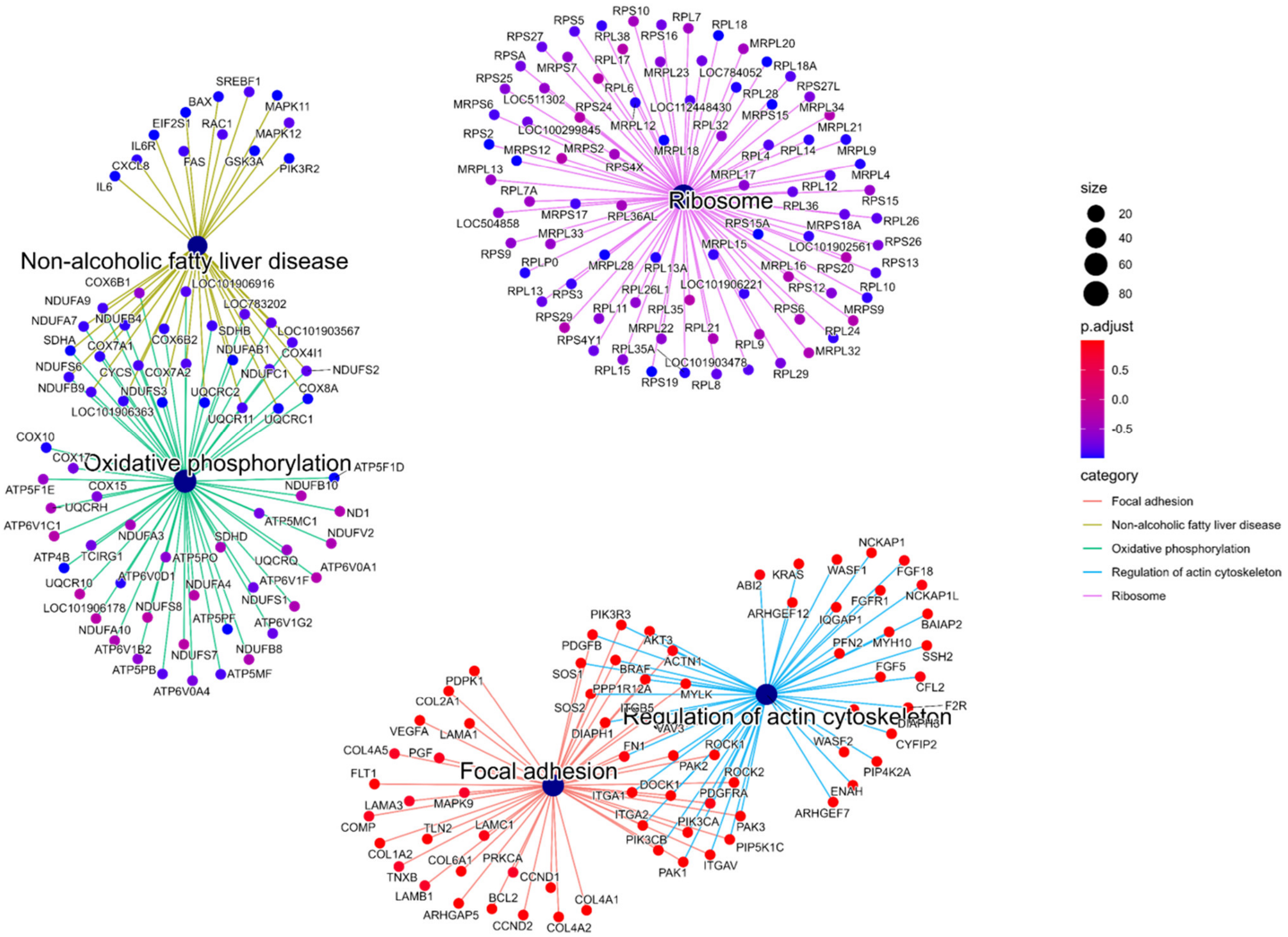

Supplementary Figure 6. GSEA analysis (KEGG pathways) following treatment with 1.8 µM AFB1. The cnetplots show the top five significantly enriched pathways. Node colors indicate the adjusted p-values (p.adjust), calculated using the BH correction method. Genes are linked to their corresponding pathways based on enrichment associations.

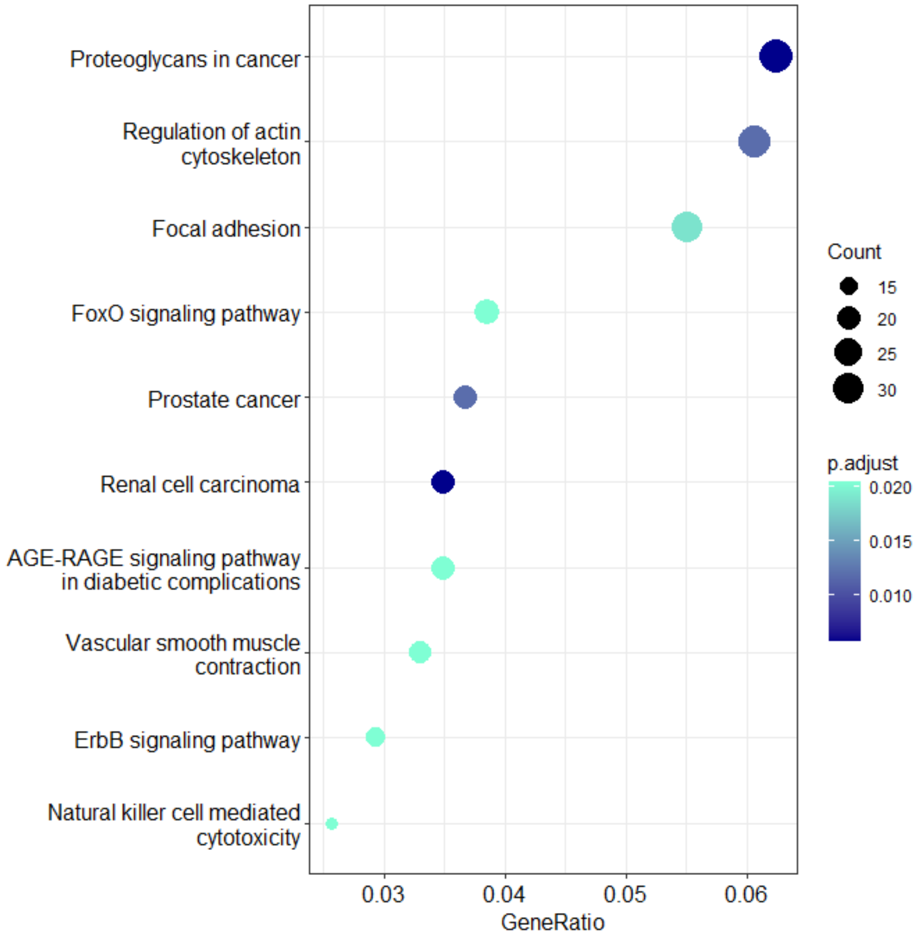

Supplementary Figure 7. KEGG overrepresentation analysis conducted on DEGs between *CYP1B1*^{KO} and *CYP1B1*^{CTL} cells incubated with AFB1. Count (dot size) represents the number of DEGs enriched in a certain pathway. The colour gradient represents the adjusted significance level (p. adjusts), according to the Benjamin-Hochberg method. The Gene Ratio represents the proportion between the number of DEGs enriched in a given pathway and the total number of genes annotated in that pathway

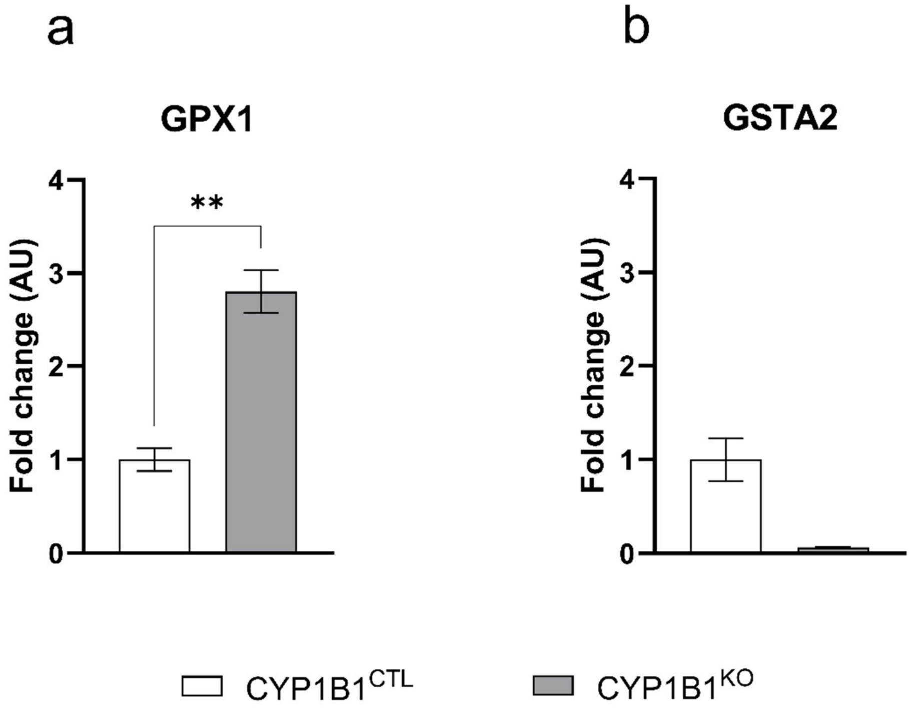

c

	RNA-seq	*qPCR*
GPX1	*1.3*	*0.5*
GSTA2	*-4.3*	*-1.2*

Supplementary Figure 8. qPCR validation of two DEGs modulated by *CYP1B1* KO. The mRNA expression of two target genes (**a, b**) was assessed by qPCR. Data are expressed as fold change (arbitrary units - AU) versus CTL, and given as mean ± SEM of three biological replicates, each analysed in duplicate. Statistical analysis: unpaired t test with Welch's correction; **: $p < 0.01$. (**c**) Comparison of RNA-seq and qPCR results in terms of logarithmic fold changes (log_2FC).

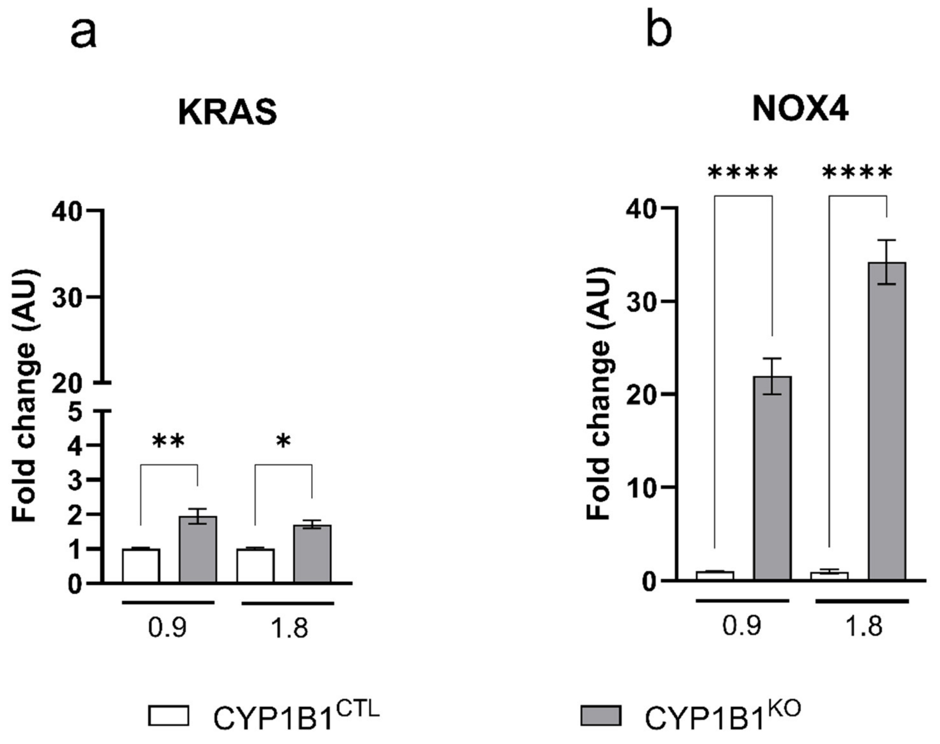

c

	RNA-seq		*qPCR*	
	0.9 µM	*1.8 µM*	*0.9 µM*	*1.8 µM*
KRAS	*1.7*	*1.9*	*0.3*	*0.2*
NOX4	*3.7*	*6.9*	*1.3*	*1.5*

Supplementary Figure 9. qPCR validation of two DEGs modulated by AFB1 (0.9 and 1.8 µM) in *CYP1B1*^{KO} cells. Data are expressed as fold change (AU) versus CTL, and given as mean ± SEM of three biological replicates, each analysed in duplicate. Statistical analysis: one-way ANOVA followed by Tukey's multiple comparisons test; *: $p < 0.05$, **: $p < 0.01$ and ****: $p < 0.0001$. (**c**) Comparison of RNA-seq and qPCR results in terms of $\log_2$FC.
